# Nausea and vomiting in pregnancy (NVP) in Chinese pregnant women: a cross-sectional study

**DOI:** 10.1186/s12884-024-06686-7

**Published:** 2024-07-16

**Authors:** Tong Zou, Zhiwen Long, Silu Wang, Qiang Yao

**Affiliations:** 1 West China Second University Hospital, Sichuan University, Chengdu, No. 20 Ren Min Nan Road, Sichuan 610041 China; 2 Key Laboratory of Birth Defects and Related Diseases of Women and Children, Sichuan University, Chengdu, Sichuan China; 3 Recovery Plus USA, New York, NY 10019 USA

**Keywords:** Nausea and vomiting in pregnancy, PUQE, RINVR, China

## Abstract

**Background:**

This study addresses the scarcity of research on nausea and vomiting in pregnancy (NVP) in China. It aims to explore the current NVP status in the country using validated questionnaires, analyze associated factors, and provide a useful reference for future research. The study also compares results from different assessment tools.

**Methods:**

Online questionnaires were utilized to gather data from 535 pregnant women across 24 provinces. Demographic, pregnancy, and NVP-related information were collected. NVP severity was assessed using Pregnancy-Unique Quantification of Emesis and Nausea (PUQE) and the Rhodes Index of Nausea, Vomiting, and Retching (RINVR) scales. Ordinal logistic regression identified factors linked to NVP severity. Differences between PUQE and RINVR assessments were compared.

**Results:**

NVP prevalence exceeded 90%, with 96.1% assessed by PUQE and 90.8% by RINVR. Incidence decreased from nausea to retching and vomiting. Severe NVP correlated with reduced gestational weight gain, younger age, fewer gestational weeks, and living in North (all *P* values < 0.05). There was moderate consistency between PUQE and RINVR assessments. The NVP prevalence assessed by the PUQE is higher than that assessed by the RINVR in the same population. However, the proportion of NVP levels above moderate assessed by RINVR is greater than that assessed by PUQE.

**Conclusions:**

NVP is highly prevalent among Chinese pregnant women, with nausea being predominant. RINVR assessments may be better able to identify severe NVP, thereby improving the low treatment rates for severe NVP.

**Supplementary Information:**

The online version contains supplementary material available at 10.1186/s12884-024-06686-7.

## Introduction

Nausea and vomiting in pregnancy (NVP) is a common complication during pregnancy, typically occurring in early pregnancy and subsiding during mid-pregnancy. However, for a minority of women, it persists into late pregnancy. Previous studies have indicated that 60–90% of pregnant women experience nausea and vomiting in pregnancy [1, 2]. NVP generally encompasses three symptoms: nausea, vomiting, and retching [3]. Although rare, there have been reports of maternal deaths or fetal demise caused by severe NVP. Severe cases, known as hyperemesis gravidarum, can lead to psychological distress and fear in pregnant women, influencing their decisions about continuing the pregnancy [4]. Additionally, severe NVP has been associated with severe clinical outcomes such as esophageal rupture, pneumothorax, Wernicke’s encephalopathy, and splenic rupture [5]. Recent studies have also identified several adverse fetal outcomes associated with severe NVP, including psychiatric disorders [6], cardiovascular diseases [7], and respiratory morbidity [8].

Research on the prevalence of NVP in China is relatively limited. Only nine articles [9–17] have reported on the current status of NVP in China, with two being in Chinese [12, 15] and seven in English [9–11, 13, 14, 16, 17]. The prevalence of NVP reported in these nine articles varies from 47 to 87.5%. Among these articles, only five specifically focused on the current status of NVP [9, 10, 12, 13, 15].

The previous studies on this topic had one or more of the following limitations:

1. The research was conducted on samples from a single city, which may not adequately represent the overall prevalence of NVP in China.
2. While some studies had a large sample size, they did not employ standardized questionnaires with good reliability and validity to assess NVP.
3. Even when reliable questionnaires like PUQE were used, the descriptions of the three distinct symptoms of NVP, including nausea, vomiting, and retching, were insufficient.

These limitations in prior research highlight the need for a more comprehensive and representative study of NVP in China, considering the diverse population and geographical variations across the country.

Currently, the commonly used questionnaire to investigate the status of NVP is PUQE. This questionnaire, recommended by the American College of Obstetricians and Gynecologists in their guidelines, consists of three questions: duration of nausea, frequency of vomiting, and frequency of retching [18]. However, PUQE assesses each of the three different symptoms using only one dimension, providing a less comprehensive description of NVP.

RINVR, developed by Rhodes, is a questionnaire that has been validated for assessing nausea and vomiting symptoms. It comprises eight questions, evaluating the duration and frequency of nausea and the level of distress caused; the frequency of vomiting, the average volume per episode, and the distress level caused; and the frequency of retching and the level of distress caused [3]. Compared to PUQE, RINVR provides a more comprehensive and detailed assessment of NVP symptoms. While RINVR is commonly used to assess postoperative or chemotherapy-induced nausea and vomiting, there has been limited research utilizing RINVR to evaluate NVP. Furthermore, there are currently no studies in China that have utilized RINVR to investigate NVP. This presents an opportunity for future research to employ RINVR for a more thorough understanding of NVP symptoms in the Chinese population.

Based on the information provided, we conducted an online questionnaire survey and utilize both PUQE and RINVR to investigate the current status of NVP across 24 of the 34 provincial-level administrative divisions in China. The objectives of this research are as follows: (1) Survey the current status of NVP in China using validated questionnaires. (2) Analyze demographic characteristics related to NVP. (3) Compare the assessment of NVP using PUQE and RINVR.

## Materials and methods

### Study design and setting

This study adopts a cross-sectional research design, conducting an online questionnaire survey to investigate the prevalence of NVP among pregnant women in China.

### Participants

This study is a sub-study of “The Effect of 5 Foods Containing Antiemetic Food Extract on the Improvement of Nausea and Vomiting of Pregnancy: A Randomized Study.” This RCT recruited participants across China using an online screening questionnaire. Participants were invited to fill out the screening questionnaire by scanning a QR code provided on an online platform. Researchers then contacted participants via the phone numbers provided in the questionnaire to verify the information (e.g., addressing incomplete responses or logical inconsistencies). Participants for this sub-study were recruited from pregnant women who completed the online screening questionnaire for the main study and whose questionnaires passed the completeness and logical consistency checks.

### Sample size

For this cross-sectional survey employing multifactor regression analysis, a sample size of 5 to 10 times the total number of study variables was targeted [19]. With 12 variables in consideration, the required sample size ranged from 60 to 120 participants. Allowing for a 10% non-response rate, 66 to 132 participants were deemed necessary.

### Variables

This study collected the following participant information: (1) General Information: Including age, height, pre-pregnancy weight, current weight, educational level and Internet Protocol (IP) address, which was used to distinguish the residence of participants. Based on the IP address, if it is located north of the Qinling-Huaihe Line, which is the geographical dividing line between northern and southern China, the residence is classified as North. If it is located south of the Qinling-Huaihe Line, the residence is classified as South. (2) Pregnancy Details: Current gestational age, number of pregnancies, number of deliveries, and fetal count. (3) Medical History: Whether participants were currently taking medications for treating NVP. (4) Assessment of NVP: Scores obtained from PUQE and RINVR evaluations.

### Data source

All variables were collected through self-administered online questionnaires completed by pregnant women. An online questionnaire was created on the Questionnaire Star platform which was opened from February 22, 2023 to February 10, 2024, and participants were recruited through various social platforms such as WeChat groups for expectant mothers and the Kangaroo Mother WeChat official account.

Participants could access and complete the questionnaire by scanning a QR code. At the end of the questionnaire, participants were required to provide their phone numbers for contact purposes in case verification of questionnaire responses was necessary.

The questionnaire’s introduction provided detailed guidance for participants as follows:

“Dear expectant mothers, please fill out the following questionnaire to preliminarily assess if you meet the inclusion criteria for our study. Please select the options that best reflect your experience of NVP. Definitions of symptoms are as follows:

Vomiting: The act of expelling stomach or intestinal contents due to discomfort.

Retching: The motion of vomiting without expelling stomach or intestinal contents.

Nausea: The feeling of sick in the upper abdomen without actual vomiting or retching.

Two researchers independently screened and checked the questionnaires. If logical errors were detected, such as an unrealistically high current weight (e.g., 150 kg), the researchers contacted the questionnaire respondents by phone to verify and correct the information. Finally, questionnaires independently checked by two researchers were cross verified. In cases of inconsistencies, respondents were contacted again for clarification.

### NVP assessment

This study employed the PUQE and RINVR questionnaires to assess NVP in pregnant women.

#### PUQE questionnaire

PUQE, recommended by the American College of Obstetricians and Gynecologists, is specifically designed to evaluate NVP. It consists of three questions: (1) On average in a day, for how long do you feel nauseated or sick to your stomach? (2) On average in a day, how many times do you vomit or throw up? (3) On average in a day, how many times have you had retching or dry heaves without bringing anything up? Each question offers five response options, scored from 1 to 5. The total PUQE score ranges from 3 to 15. A score of 3 indicates no NVP, scores > 3 and ≤ 6 represent mild NVP, scores > 6 and ≤ 12 indicate moderate NVP, and scores ≥ 13 indicate severe NVP.

#### RINVR questionnaire

RINVR, developed by Rhodes and validated for reliability and validity, assesses immediate experiences of nausea and vomiting, such as those occurring within the past 24 h. RINVR comprises eight questions across three dimensions: nausea, vomiting, and retching/dry heaves. The questions include details such as the number of episodes of vomiting and retching, the level of distress experienced, and the duration of symptoms. Each question has five response options, scored from 0 to 4. The total RINVR score ranges from 0 to 32. A score of 0 indicates no NVP, scores from 1 to 8 represent mild NVP, scores from 9 to 16 indicate moderate NVP, scores from 17 to 24 represent severe NVP, and scores from 25 to 32 indicate extremely severe symptoms.

### Statistical analysis

All statistical analyses were performed using SAS 9.4 (SAS Institute Inc., Cary, NC, USA). Normality tests revealed non-normal distribution of all quantitative variables. Hence, median and interquartile range (IQR) were used for describing quantitative variables. Categorical variables were described using frequencies and percentages.

Comparisons between groups for quantitative variables were conducted using the Mann-Whitney U test, while chi-square tests were employed for categorical variables. The relationship between demographic/periodic features and NVP was analyzed through ordinal logistic regression. PUQE-assessed NVP severity and RINVR-assessed NVP severity were used as dependent variables, and demographic/periodic features were used as independent variables. Initial univariate analyses were performed, and variables showing statistical significance were included in the multivariate analysis.

As PUQE categorized NVP into 4 levels and RINVR into 5 levels, RINVR categories were consolidated into 4 levels (no NVP, mild NVP, moderate NVP, and severe NVP, with ‘severe’ combining the original ‘great’ and ‘severe’ categories) for comparison with PUQE. Discrepancies between PUQE and RINVR assessments were analyzed using the CMH chi-square test and Kappa consistency test. A significance level of *P* < 0.05 was considered statistically significant.

## Results

### Participants

A total of 562 pregnant women completed the survey questionnaires. Among them, 27 questionnaires had issues and could not be verified due to incorrect or missing phone numbers. Consequently, 535 questionnaires were included in the final analysis.

### General characteristics of participants

The median age of the participants was 29 years (IQR 27 − 32), with a median gestational age (GW) of 12.1 weeks (IQR 9.4 − 14.7). The median pre-pregnancy BMI was 21 kg/m² (IQR 19.5 − 23.4), with the median gestational weight gain (GWG) of 1 kg (IQR 0 − 2) (Table 1).

Most participants were carrying a single fetus (97.9%), were living in Southern China (87.9%), had a bachelor’s degree or higher education level (83.7%), were in early pregnancy (72.7%), had no history of previous deliveries (57.5%), and were first-time pregnant (56.8%).

**Table 1:** General characteristic of participants

Variables		
Age, in years, median (IQR^a^)		29 (27 − 32)
Pre-pregnancy BMI^b^, in kg/m^2^, median (IQR)		21.0 (19.5 − 23.4)
BMI, in kg/m^2^, median (IQR)		21.5 (19.6 − 23.6)
Pre-pregnancy BMI category, n (%)	Underweight	99 (18.5)
	Normal weight	338 (63.2)
	Overweight	68 (12.7)
	Obesity	30 (5.6)
GWG^c^, in kg, median (IQR)		1 (0 − 2)
GW^d^, in weeks, median (IQR)		12.1 (9.4 − 14.7)
GW, n (%)	< 14 weeks	389 (72.7)
	≥ 14 weeks	146 (27.3)
Gestational times, n (%)	1	304 (56.8)
	> 1	231 (43.2)
Parity times, n (%)	0	289 (57.5)
	≥ 1	214 (42.5)
Education, n (%)	Junior high school	40 (8.0)
	Senior high school	42 (8.3)
	College or university	358 (71.2)
	Postgraduate	63 (12.5)
Number of fetuses, n (%)	Single	524 (97.9)
	Multiple	11 (2.1)
Whether taking treatment, n (%)	Yes	33 (6.2)
	NO	502 (93.8)
Residence, n (%)	South	470 (87.9)
	North	65 (12.1)

^a^ IQR, inter quartile range; ^b^ BMI, body mass index; ^c^ GWG, gestational weight gain; ^d^ GW, gestational week

### NVP prevalence in Chinese pregnant women

#### NVP prevalence assessed by PUQE

As shown in Table 2, the median PUQE score among participants was 8 (IQR 6 − 10). Most pregnant women experienced NVP (96.1%). 21.5% had mild NVP, 67.5% had moderate NVP, and 7.1% had severe NVP. The rates of nausea, retching, and vomiting decreased in sequence, with rates of 95.7%, 85.4%, and 73.1%, respectively.

**Table 2:** NVP^a^ assessment using PUQE^b^ and RINVR^c^

Questionnaire			
PUQE	Total score, in median (IQR^d^)		8 (6 − 10)
	Nausea prevalence, in n (%)		512 (95.7)
	Retching prevalence, in n (%)		457 (85.4)
	Vomiting prevalence, in n (%)		391 (73.1)
	NVP degree, in n (%)	None	21 (3.9)
		Mild	115 (21.5)
		Moderate	361 (67.5)
		Severe	38 (7.1)
RINVR	Total score, median (IQR)		13 (7 − 18)
	Nausea prevalence, in n (%)		479 (89.5)
	Retching prevalence, in n (%)		421 (78.7)
	Vomiting prevalence, in n (%)		343 (64.1)
	NVP degree, in n (%)	None	49 (9.2)
		Mild	120 (22.4)
		Moderate	194 (36.3)
		Great	145 (27.1)
		Severe	27 (5.0)

^a^ NVP, nausea and vomiting during pregnancy. ^b^ PUQE, Pregnancy-Unique Quantification of Emesis and Nausea. ^c^ RINVR, the Rhodes Index of Nausea, Vomiting and Retching. ^d^ IQR, inter quartile range

#### NVP degree assessed by RINVR as dependent variables

As indicated in Table 2, the median RINVR score for participants was 13 (IQR 7 − 18). Most pregnant women experienced NVP (90.8%). 22.4% had mild NVP, 36.3% had moderate NVP, 27.1% had significant NVP, and 5% had severe NVP. Similar to the PUQE assessment, the incidence rates of nausea, retching, and vomiting decreased sequentially to 89.5%, 78.7%, and 64.1%, respectively. The distress caused by nausea and retching was higher than vomiting, with median distress scores being nausea: retching: vomiting = 2:2:1.

### Univariate analysis of factors influencing NVP

#### NVP degree assessed by PUQE as dependent variables

As indicated in Table 3, pregnant women with GW less than 14 weeks (*P* = 0.0001) and those who took treatment measures (*P* = 0.001) experienced higher severity of NVP. Pregnant women with more severe NVP had lesser GWG (*P* < 0.0001). There were no statistically significant differences in the severity of NVP based on age, pre-pregnancy BMI, gestational times, parity times, education level, number of fetuses, or residence.

**Table 3:** NVP^a^ degree assessed by PUQE^b^ between different groups

Variables			NVP degree			*P* value
		None	Mild	Moderate	Severe	
Age, n (%)	< 35 years	15 (3.1)	102 (21.4)	323 (67.9)	36 (7.6)	0.077
	≥ 35 years	6 (10.2)	13 (22)	38 (64.4)	2 (3.4)	
GW^c^, n (%)	< 14 weeks	13 (3.3)	68 (17.5)	276 (71)	32 (8.2)	0.0001
	≥ 14 weeks	8 (5.5)	47 (32.2)	85 (58.2)	6 (4.1)	
Pre-pregnancy BMI^d^, n (%)	Underweight	2 (2)	24 (24.2)	67 (67.7)	6 (6.1)	0.809
	Normal weight	15 (4.4)	67 (19.8)	233 (68.9)	23 (6.8)	
	Overweight	3 (4.4)	20 (29.4)	37 (54.4)	8 (11.8)	
	Obesity	1 (3.3)	4 (13.3)	24 (80)	1 (3.3)	
GWG^e^, median (IQR^f^)		2 (0 − 4.5)	1 (0 − 3)	0.5 (-0.5 − 2)	0 (-2.5 − 1)	< 0.0001
Gestational times, n (%)	1	15 (4.9)	69 (22.7)	200 (65.8)	20 (6.6)	0.146
	> 1	6 (2.6)	46 (19.9)	161 (69.7)	18 (7.8)	
Parity times, n (%)	0	11 (3.8)	71 (24.6)	189 (65.4)	18 (6.2)	0.134
	≥ 1	9 (4.2)	39 (18.2)	149 (69.6)	17 (7.9)	
Education, n (%)	Junior high school	1 (2.5)	7 (17.5)	29 (72.5)	3 (7.5)	0.096
	Senior high school	1 (2.4)	5 (11.9)	32 (76.2)	4 (9.5)	
	College or university	17 (4.8)	78 (21.8)	236 (65.9)	27 (7.5)	
	Postgraduate	1 (1.6)	20 (31.7)	41 (65.1)	1 (1.6)	
Number of fetuses, n (%)	Single	21 (4)	115 (22)	350 (66.8)	038 (7.2)	0.198
	Multiple	0 (0)	0 (0)	11 (100)	0 (0)	
Residence, n (%)	South	21 (4.5)	101 (21.5)	317 (67.4)	31 (6.6)	0.195
	North	0 (0)	14 (21.5)	44 (67.7)	7 (10.8)	
Whether taking treatment, n (%)	Yes	0 (0)	2 (6.1)	25 (75.8)	6 (18.2)	0.001
	No	21 (4.2)	113 (22.5)	336 (66.9)	32 (6.4)	

^a^ NVP, nausea and vomiting during pregnancy. ^b^ PUQE, Pregnancy-Unique Quantification of Emesis and Nausea. ^c^ GW, gestational week; ^d^ BMI, body mass index; ^e^ GWG, gestational week gain. ^f^ IQR, inter quartile range

#### NVP degree assessed by RINVR as dependent variables

As shown in Table 4, pregnant women under the age of 35 (*P* = 0.001), those with GW less than 14 weeks (*P* < 0.0001), residing in the northern regions (*P* = 0.003), and those taking treatment measures (*P* = 0.009) experienced higher severity of NVP. Pregnant women with more severe NVP had lesser GWG (*P* < 0.0001). No significant correlations were found between NVP severity and other factors.

**Table 4:** NVP^a^ degree assessed by RINVR^b^ between different groups

Variables			NVP degree				*P* value
		None	Mild	Moderate	Great	Severe	
Age, n (%)	< 35 years	37 (7.8)	104 (21.8)	174 (36.5)	136 (28.6)	25 (5.3)	0.001
	≥ 35 years	12 (20.3)	16 (27.1)	20 (33.9)	9 (15.3)	2 (3.4)	
GW^c^, n (%)	< 14 weeks	26 (6.7)	79 (20.3)	145 (37.3)	117 (30.1)	22 (5.7)	< 0.0001
	≥ 14 weeks	23 (15.8)	41 (28.1)	49 (33.6)	28 (19.2)	5 (3.4)	
Pre-pregnancy BMI^d^, n (%)	Underweight	6 (6.1)	20 (20.2)	37 (37.4)	32 (32.3)	4 (4)	0.364
	Normal weight	33 (9.8)	77 (22.8)	117 (34.6)	89 (26.3)	22 (6.5)	
	Overweight	7 (10.3)	20 (29.4)	22 (32.4)	18 (26.5)	1 (1.5)	
	Obesity	3 (10)	3 (10)	18 (60)	6 (20)	0 (0)	
GWG^e^, median (IQR ^f^)		2 (0 − 4)	1 (0 − 3)	1 (0 − 2.4)	0 (-1.5 − 1)	0 (-1 − 0.5)	< 0.0001
Gestational times, n (%)	1	32 (10.5)	68 (22.4)	106 (34.9)	82 (27)	4 (5.3)	0.607
	> 1	17 (7.4)	52 (22.5)	88 (38.1)	63 (27.3)	11 (4.8)	
Parity times, n (%)	0	31 (10.7)	70 (24.2)	101 (35)	73 (25.3)	14 (4.8)	0.23
	≥ 1	18 (8.4)	45 (21)	80 (37.4)	60 (28)	11 (5.1)	
Education, n (%)	Junior high school	4 (10)	6 (15)	22 (55)	7 (17.5)	1 (2.5)	0.402
	Senior high school	3 (7.1)	5 (11.9)	17 (40.5)	17 (40.5)	0 (0)	
	College or university	38 (10.6)	83 (23.2)	122 (34.1)	94 (26.3)	21 (5.9)	
	Postgraduate	4 (6.4)	21 (33.3)	20 (31.7)	15 (23.8)	3 (4.8)	
Number of fetuses, n (%)	Multiple	0 (0)	1 (9.1)	4 (36.4)	6 (54.6)	0 (0)	0.083
	Single	49 (9.4)	119 (22.7)	190 (36.3)	139 (26.5)	27 (5.1)	
Residence, n (%)	South	48 (10.2)	108 (23)	172 (36.6)	120 (25.5)	22 (4.7)	0.003
	North	1 (1.5)	12 (18.5)	22 (33.9)	25 (38.5)	5 (7.7)	
Whether taking treatment, n (%)	Yes	47 (9.4)	115 (22.9)	186 (37)	132 (26.3)	22 (4.4)	0.009
	No	2 (6.1)	5 (15.2)	8 (24.2)	13 (39.4)	5 (15.1)	

^a^ NVP, nausea and vomiting during pregnancy. ^b^ RINVR, the Rhodes Index of Nausea, Vomiting and Retching; ^c^ GW, gestational week; ^d^ BMI, body mass index; ^e^ GWG, gestational week gain. ^f^ IQR, inter quartile range

### Multivariate analysis of factors related to NVP

#### PUQE assessment results as dependent variables

As shown in Table 5, after controlling for GW and treatment measures, pregnant women with severe NVP had lesser GWG compared to those without NVP (*P* = 0.002).

**Table 5:** Multiple logistic regression for NVP^a^ degree assessed by PUQE^b^

Variables	NVP degree	DF	Estimate	Standard error	Wald	*P* value
GW^c^ is > or = 14 weeks vs. GW is < 14 weeks	None	Ref.				
	Mild	1	0.2495	0.5326	0.2195	0.6394
	Moderate	1	-0.4862	0.5101	0.9085	0.3405
	Severe	1	-0.6511	0.6752	0.9301	0.3348
Taking treatment vs. not taking treatment	None	Ref.				
	Mild	1	10.9646	401.6	0.0007	0.9782
	Moderate	1	12.2780	401.6	0.0009	0.9756
	Severe	1	13.0690	401.6	0.0011	0.9740
GWG^d^	None	Ref.				
	Mild	1	-0.0469	0.0643	0.5315	0.4660
	Moderate	1	-0.0631	0.0616	1.0508	0.3053
	Severe	1	-0.2884	0.0935	9.5112	0.0020

^a^ NVP, nausea and vomiting during pregnancy. ^b^ PUQE, Pregnancy-Unique Quantification of Emesis and Nausea. ^c^ GW, gestational week; ^d^ GWG, gestational week gain.

#### RINVR assessment results as dependent variables

As depicted in Table 6, older age and later gestational age, as well as residing in southern China, were independently associated with lower severity of NVP. Pregnant women with severe and great NVP experienced lesser weight gain during pregnancy.

**Table 6:** Multiple logistic regression for NVP^a^ degree assessed by RINVR^b^

Variables	NVP degree	DF	Estimate	Standard error	Wald	*P* value
Age > or = 35 years vs. Age < 35 years	None	Ref.				
	Mild	1	-0.7291	0.4351	2.8078	0.0938
	Moderate	1	-1.0131	0.4202	5.8130	0.0159
	Great	1	-1.5835	0.5041	9.8681	0.0017
	Severe	1	-1.5013	0.8427	3.1740	0.0748
GW^c^ is > or = 14 weeks vs. GW is < 14 weeks	None	Ref.				
	Mild	1	-0.5352	0.3722	2.0681	0.1504
	Moderate	1	-0.9467	0.3583	6.9812	0.0082
	Great	1	-1.2441	0.3922	10.0622	0.0015
	Severe	1	-1.1957	0.6178	3.7454	0.0530
Taking treatment vs. not taking treatment	None	Ref.				
	Mild	1	0.0657	0.8614	0.0058	0.9392
	Moderate	1	0.0594	0.8187	0.0053	0.9422
	Great	1	0.8419	0.8051	1.0935	0.2957
	Severe	1	1.7491	0.9094	3.6997	0.0544
GWG^d^	None	Ref.				
	Mild	1	-0.0247	0.0524	0.2229	0.6368
	Moderate	1	-0.0387	0.0507	0.5830	0.4451
	Great	1	-0.1412	0.0569	6.1517	0.0131
	Severe	1	-0.2257	0.0931	5.8845	0.0153
Living in South China vs. living in North China	None	Ref.				
	Mild	1	-1.6053	1.1065	2.1048	0.1468
	Moderate	1	-1.9225	1.0834	3.1486	0.0760
	Great	1	-3.0363	1.0888	7.7773	0.0053
	Severe	1	-3.3358	1.2094	7.6078	0.0058

^a^ NVP, nausea and vomiting during pregnancy. ^b^ RINVR, the Rhodes Index of Nausea, Vomiting and Retching; ^c^ GW, gestational week; ^d^ GWG, gestational week gain

### Comparison of PUQE and RINVR assessments for NVP

As presented in Table 7, there is a significant correlation between the NVP severity assessed by PUQE and RINVR (χ² ^CMH^ = 244, *P* < 0.001). The assessments from both methods demonstrate moderate consistency (Weighted Kappa = 0.46, 95% *CI*: 0.41, 0.51).

**Table 7:** Comparison of NVP^a^ degree assessed by PUQE^b^ and RINVR^c^

	NVP degree assessed by RINVR, *n* (%)					
		None	Mild	Moderate	Severe	Total
NVP degree assessed by PUQE	None	20 (3.7)	1 (0.2)	0 (0)	0 (0)	21 (3.9)
	Mild	22 (4.1)	67 (12.5)	25 (4.7)	1 (0.2)	115 (21.5)
	Moderate	7 (1.3)	51 (9.5)	167 (31.2)	136 (25.4)	361 (67.5)
	Severe	0 (0)	1 (0.2)	2 (0.4)	35 (6.5)	38 (7.1)
	Total	49 (9.2)	120 (22.4)	194 (36.3)	172 (32.2)	535 (100)

^a^ NVP, nausea and vomiting during pregnancy. ^b^ PUQE, Pregnancy-Unique Quantification of Emesis and Nausea. ^c^ RINVR, the Rhodes Index of Nausea, Vomiting and Retching

The data in the table is represented as: n (%)

χ² _CMH_ = 244, *P* < 0.001

Weighted Kappa = 0.46 (95% CI: 0.41, 0.51)

## Discussion

### Prevalence of NVP in Chinese pregnant women

This study demonstrates that regardless of whether the PUQE or RINVR assessment methods were employed, the prevalence rate of NVP in Chinese pregnant women exceeded 90%. This is close to the 90.9% [20] reported by Oi Ka Chan and colleagues, slightly higher than the 84.1–87.5% reported in other Chinese studies [9, 17]. Several factors might contribute to this variance. First, the diverse assessment methods used in different studies could lead to varied prevalence rates. This study utilized validated and standardized PUQE and RINVR questionnaires. Second, the sample in this study represented 24 provinces in China, enhancing its representativeness compared to previous studies.

The prevalence of NVP among Chinese pregnant women, as indicated by this study, is also higher than that reported in Spain (63.5%) [21], Canada (63.3%) [22], and the United States (68.6%) [23]. Additionally, the high prevalence of NVP in China may be related to the low smoking rates among Chinese women. Research indicates that pre-pregnancy smoking can reduce the risk of NVP by increasing growth differentiation factor 15 (GDF15) levels, whereas non-smokers typically have lower GDF15 levels [24–26]. GDF15 is correlated with NVP, interacting with the Glial-derived neurotrophic factor receptor alpha-like (GFRAL) protein, a key pathogenic mechanism of NVP [27]. Pre-pregnancy exposure to higher levels of GDF15 can desensitize women to the rapid increase in GDF15 during early pregnancy, thereby alleviating NVP symptoms [28]. According to a study, the smoking rate among Chinese women remained between 2.2% and 1.9% from 2007 to 2018 [29]. Another study reported that the smoking rate among Chinese women was about 3.4% in 2010, indicating a generally low smoking trend [30].Therefore, the low smoking rate among Chinese women may help explain the higher prevalence of NVP in China.

### Different NVP symptoms among Chinese pregnant women

When considering different NVP symptoms, the frequency of nausea, retching, and vomiting among Chinese pregnant women decreases in that order. According to the RINVR assessment results, the distress caused by nausea and retching is stronger than that caused by vomiting, possibly due to the higher frequency of nausea and retching. Vomiting represents the final symptom in the progression of NVP, involving a complex reflex action that can be divided into three stages: nausea, retching, and vomiting [3]. During nausea, gastric tension and peristalsis weaken, while duodenal tension increases, possibly accompanied by duodenal reflux. Retching involves relaxation of the upper part of the stomach and brief contraction of the gastric antrum. Vomiting occurs when the gastric antrum continues to contract, the lower esophageal sphincter relaxes, abdominal muscles contract, the diaphragm descends, abdominal pressure increases, forcing gastric contents to rapidly and violently reflux from the stomach, through the esophagus and mouth, and out of the body [31]. Recent research has found that GDF15 derived from the fetoplacental unit is highly likely to be the cause of NVP. Studies have shown that in early pregnancy, the levels of GDF15 in maternal blood appear alongside NVP symptoms and show a strong correlation with these symptoms. Whole exome sequencing has identified GDF15 as the strongest risk factor associated with severe NVP [32]. Furthermore, GDF15 has also been shown to be related to symptoms such as loss of appetite, taste aversion, nausea, vomiting, and weight loss [33–38]. The latest research continues to support that GDF15 derived from the fetoplacental unit is highly likely to be a causal factor of NVP [28]. HCG was previously thought to be associated with the occurrence and severity of NVP, but genetic studies did not find any association with the hCG gene or receptor [32].

### Factors related to NVP

Multivariate analysis revealed a consistent finding with other studies: more severe NVP correlates with lesser weight gain during pregnancy, a phenomenon noted in previous research as well [21, 39, 40]. Symptoms like nausea, vomiting, and retching may diminish a pregnant woman’s appetite, leading to reduced food intake and digestion, consequently resulting in lower weight gain during pregnancy [41].

This study also found that older age and later gestational weeks are associated with lower severity of NVP, aligning with similar findings in other research studies [20, 42]. Previous studies have found a correlation between multiple pregnancies and the severity of NVP [42]. However, our study did not find a statistically significant association between multiple pregnancies and NVP. This may be due to the small sample size, as our study included only 11 cases of multiple pregnancies, and the NVP severity in these 11 cases was all rated as ‘Moderate’ by PUQE. To explore the relationship between multiple pregnancies and NVP further, future research should include a larger sample size of multiple pregnancies.

This study is the first to compare the severity of NVP between Northern and Southern China. In a multifactorial analysis using the RINVR to assess NVP severity, we found that the rate of severe NVP was lower in Southern China compared to Northern China. Since no previous studies directly analyzed the differences in NVP severity between the North and South, we reviewed literature investigating the prevalence of NVP in China. We identified three studies [10, 13, 43] with settings that could be categorized as either Northern or Southern China. Two studies [13, 43] conducted in Southern China had a combined sample size of 4,654 participants, with 2,277 (48.9%) experiencing mild NVP, 1,229 (26.4%) experiencing moderate NVP, and 61 (1.3%) experiencing severe NVP. One study used the PUQE questionnaire [13], while the other used a self-developed questionnaire [43]. One study conducted in Northern China [10] had a sample size of 3,064 participants, with 299 (9.8%) experiencing severe NVP, assessed by asking participants, “Have you experienced severe nausea and vomiting during early pregnancy accompanied by urinary ketone positivity? (Yes/No).” Severe NVP was defined as persistent or aggravated nausea and vomiting in early pregnancy, accompanied by ketonuria. These studies indicate a higher incidence of severe NVP in Northern China, consistent with our findings that Northern patients have a higher prevalence of severe NVP.

We further classified patients into six regions based on the Chinese six-region division method and their IP addresses: Central-South China, East China, North China, Northeast China, Northwest China, and Southwest China. We then grouped the patients and calculated the percentage of “Severe” and “Great” NVP levels assessed by the RINVR questionnaire in each region. The percentages from highest to lowest were East China (27 [54%]), North China (52.7%), Northwest China (5 [45.5%]), Central-South China (20 [35.5%]), Southwest China (137 [34.9%]), and Northeast China (1 [25%]). This analysis revealed that the combined percentages of “Severe” and “Great” levels exceeded 40% in East China, North China, and Northwest China, while they were below 40% in Central-South China, Southwest China, and Northeast China. Due to the sample size limitations, further analysis at the provincial or municipal level was not feasible.

China’s vast territory encompasses diverse geographic and climatic characteristics, dietary habits, and perceptions of NVP. The differences in NVP severity between Northern and Southern China may be related to these geographic, climatic, and cultural factors, as well as differing attitudes toward NVP symptoms. Our study suggests that Northern pregnant women and obstetric healthcare providers should pay closer attention to NVP due to its association with adverse maternal and fetal outcomes.

In summary, this is an interesting finding that warrants further research specifically on the differences in NVP between Northern and Southern China. Future studies should collect detailed information on NVP, diet, physical activity, and perceptions of NVP among patients and their families to explore potential factors contributing to the differences in NVP severity between the regions.

### Comparison of NVP assessment between PUQE and RINVR

Both PUQE and RINVR were proved validated for assessing NVP in previous studies [44, 45]. This study found a moderate consistency between the NVP severity assessed by PUQE and RINVR, indicating some differences.

Firstly, the NVP prevalence assessed by the PUQE is higher than that assessed by the RINVR in the same population. This discrepancy may arise because the two questionnaires evaluate different time spans. PUQE assesses the NVP condition over the entire pregnancy up to the point of filling out the questionnaire [46, 47], while RINVR assesses the patient’s condition over the past 24 h at the time of questionnaire completion [48]. The frequency and severity of NVP symptoms may fluctuate throughout pregnancy, especially after the first trimester (27.3% of the patients in this study were beyond 14 weeks of gestation) [49]. It is possible that the symptoms had eased or were absent the day before the patient filled out the RINVR, leading to a lower prevalence and severity level compared to PUQE, even though RINVR includes more items.

Secondly, after combining the “Great” and “Severe” levels assessed by RINVR, the combined “Severe” rate is actually higher than the “Severe” rate assessed by PUQE. This might be due to the same reason as the first issue, i.e., the different time spans assessed by PUQE and RINVR. Additionally, the difference in NVP severity classification between PUQE and RINVR might contribute to this discrepancy. PUQE classifies NVP into four levels: None, Mild, Moderate, and Severe. In contrast, RINVR divides NVP into five levels: None, Mild, Moderate, Great, and Severe. In this study, among the patients classified as “Great” by RINVR (145 patients), 20 were classified as “Severe” (PUQE score ≥ 13), 124 as “Moderate” (PUQE score 7–12), and 1 as “Mild” (PUQE score 4–6) according to PUQE. These data suggest that most patients rated as “Great” by RINVR might fall into the “Moderate” category according to PUQE, with a smaller portion falling into the “Severe” category. This indicates that using PUQE may lead to some underdiagnosis and undertreatment. As previously mentioned, severe NVP is associated with adverse pregnancy and fetal outcomes. Therefore, RINVR may be a more suitable tool for improving these outcomes. Additionally, RINVR assesses a broader range of symptoms, including the distress caused by nausea, retching, and vomiting, providing a more comprehensive understanding of NVP experiences.

## Strengths and limitations

This study has several limitations. First, this study was conducted through an online self-administered questionnaire, which may result in selection bias, because pregnant women who have NVP-related symptoms are more willing to fill in the questionnaire. This may also be one of the reasons why this study found that the prevalence of NVP in China is higher. Second, this study did not conduct stratified sampling according to different regions in China, which may limit the representativeness of the sample in this study. However, this study is the first nationwide NVP survey in China, involving 24 provinces/autonomous regions/municipalities, and the sample is still representative of China. In addition, this study found differences in the prevalence and severity of NVP between northern and southern China, which guides subsequent studies to further study the differences in NVP in different regions of China, and has guiding significance for formulating corresponding NVP intervention strategies for different regions in China. Finally, this study was the first to compare the differences between PUQE and RINVR in assessing NVP levels in China and found that PUQE may underestimate the degree of NVP in patients. Considering that severe NVP is associated with adverse outcomes for both mothers and fetuses, we suggest that regular assessment using RINVR may be better. Additionally, one limitation of our study is that the PUQE and RINVR tools assess symptoms over different time spans, which might contribute to the differences observed in the results. Further studies are needed to compare the two tools covering the same time span in China.

## Conclusions

This cross-sectional study revealed a high prevalence of NVP among Chinese pregnant women, with age, gestational age, living in South and gestational weight gain showing a negative correlation with the severity of NVP. These findings enhance our understanding of NVP in the Chinese population and provide a useful reference for future research. Both PUQE and RINVR prove useful for NVP assessment. However, RINVR assessments may be better able to identify severe NVP, thereby improving the low treatment rates for severe NVP.

### Electronic supplementary material

Below is the link to the electronic supplementary material.

Supplementary Material 1

Supplementary Material 2

## Data Availability

The datasets generated and analyzed during the current study are not publicly available due privacy and ethical reasons, but are available from the corresponding author on reasonable request.
