# Supplementary material for: Nausea and vomiting in pregnancy (NVP) in Chinese pregnant women: a cross-sectional study
Source: BMC Pregnancy Childbirth. 2024 Jul 16;24:481. doi: 10.1186/s12884-024-06686-7 (PMC11251098; doi:10.1186/s12884-024-06686-7)
Supplement: Supplementary file 1 — Supplementary Material 1 [file 12884_2024_6686_MOESM1_ESM.pdf]

PUQE 问卷

1. 一天当中，您感觉胃部不适或恶心的时间有多久?
   从未 （1）
   1 小时或更少（2）
   2-3 小时（3）
   4-6 小时（4）
   ≥6 小时（5）
2. 一天当中，您会呕吐几次?
   ≥7 次（5） 5-6 次（4） 3-4 次（3） 1-2 次（2） 从未（1）
3. 一天当中，您会干呕（没有内容物、没吐出东西）几次?
   从未（1） 1-2 次（2） 3-4 次（3） 5-6 次（4） ≥7 次（5）

总分为 3 题相加：轻度，≤6 分；中度，7-12 分；重度，≥13 分

RINVR 问卷

1. 过去的 24 小时中，我吐了几次。
   >7 次 (4) 5-6 次 (3) 3-4 次 (2) 1-2 次 (1) 没有吐 (0)
2. 在过去的 24 小时中，因为干呕，我觉得（ ）不舒服
   没有 (0) 一点 (1) 中等程度 (2) 十分 (3) 非常严重 (4)
3. 在过去的 24 小时中，因为呕吐，我觉得（）不舒服
   非常严重 (4) 十分 (3) 中等程度 (2) 一点 (1) 没有 (0)
4. 过去的 24 小时中，胃里恶心的感觉持续多久?
   没有 (0) ≤1 小时 (1) 2-3 小时 (2) 4-6 小时 (3) >6 小时 (4)
5. 在过去的 24 小时中，因为恶心，我觉（）不舒服
   没有 (0) 一点 (1) 中等程度 (2) 十分 (3) 非常严重 (4)
6. 过去的 24 小时中，我每次呕吐的量大约有多少?
   很多 (>750 ml) 多 (500-750 ml) 中等 (125-500ml) 少 (<125 ml） 没有吐
7. 在过去的 24 小时中，我感觉恶心几次?
   >7 次 (4) 5-6 次 (3) 3-4 次 (2) 1-2 次 (1) 没有 (0)
8. 过去的 24 小时中，我干呕（想吐却吐不出来）几次?
   没有 (0) 1-2 次 (1) 3-4 次 (2) 5-6 次 (3) >7 次 (4)

总分为 8 题相加，分数范围为 0-32 分。根据总分分级如下：无 NVP，0 分；轻度，1-8 分；中度，9-16 分；重度，17-24 分；非常严重，25-32 分。

恶心评分为第 4、5、7 题分数相加，分数范围为 0-12 分；呕吐评分为第 1、3、6 题分数相加，分数范围为 0-12 分；干呕评分为第 2、8 题相加，分数范围为 0-8 分。
