# Supplementary material for: Nausea and vomiting in pregnancy (NVP) in Chinese pregnant women: a cross-sectional study
Source: BMC Pregnancy Childbirth. 2024 Jul 16;24:481. doi: 10.1186/s12884-024-06686-7 (PMC11251098; doi:10.1186/s12884-024-06686-7)
Supplement: Supplementary file 2 — Supplementary Material 2 [file 12884_2024_6686_MOESM2_ESM.pdf]

筛查问卷

孕妈妈您好！我们正在开展华西公共卫生学院和 a1 零食研究所联合发起的题目为【含抑吐植提物的 5 种食品对孕期恶心、呕吐、干呕症状的改善作用研究】的研究项目。请您填写以下问卷以初步筛查您是否符合本研究的入组标准。

请注意：请选择**最能够反应**您妊娠期恶心呕吐情况的选项。各症状定义如下：
呕吐：因为不舒服吐出了胃肠道内容物（吐了东西出来）；
干呕：有呕吐的动作但是没有吐出胃肠道内容物；
恶心：指上腹部有不舒服的感觉，不会出现呕吐或干呕的动作。

您当前年龄（岁）：[填空题] *

____

您的身高是（m）：[填空题] *

____

您当前体重是（kg）：[填空题] *

____

您孕前体重是（kg）：[填空题] *

____

您当前孕周是（例：12+3 周）： [填空题] *

____

怀孕次数： [填空题] *

____

生产次数： [填空题] *

____

文化程度： [单选题] *

○小学

○初中

○高中或职中

○大专或本科

○研究生

1. 一天当中，您感觉胃部不适或恶心的时间有多久?（一共累计的时间） [单选题]

○从未 ○1 小时或更少 ○2-3 小时 ○4-6 小时 ○≥6 小时

2. 一天当中，您会呕吐几次（吐出了胃内容物）? [单选题] *

○≥7 次 ○5-6 次 ○3-4 次 ○1-2 次 ○从未

3. 一天当中，您会干呕（没有内容物、没吐出东西）几次? [单选题] *

○从未 ○1-2 次 ○3-4 次 ○5-6 次 ○≥7 次

4. 过去的 24 小时中，我吐了几次? [单选题] *

○>7 次　○5-6 次　○3-4 次　○1-2 次　○没有吐

5.在过去的 24 小时中，因为干呕，我觉得（ ）不舒服 [单选题] *

○没有　○一点　○中等程度　○十分　○非常严重

6.在过去的 24 小时中，因为呕吐，我觉得（ ）不舒服 [单选题] *

○非常严重　○十分　○中等程度　○一点　○没有

7.过去的 24 小时中，我胃里恶心的感觉持续多久?（一共累计的时间） [单选题] *

○没有　○≤1 小时　○2-3 小时　○4-6 小时　○>6 小时

8.在过去的 24 小时中，因为恶心，我觉（ ）不舒服 [单选题] *

○没有　○一点　○中等程度　○十分　○非常严重

9.过去的 24 小时中，我每次呕吐的量大约有多少? [单选题] *

○很多（>750 ml）　○多（500-750 ml）　○中等（125-500ml）　○少（<125 ml）
○没有吐

10.在过去的 24 小时中，我感觉恶心几次? [单选题] *

○>7 次　○5-6 次　○3-4 次　○1-2 次　○没有

11.过去的 24 小时中，我干呕（想吐却吐不出来）几次? [单选题] *

○没有　○1-2 次　○3-4 次　○5-6 次　○>7 次

12. 您的年龄在 18~40 岁？ [单选题] *

○是

○否

13. 您目前的孕周是？ [单选题] *

○＜20 周

○≥20 周

14. 您怀的是单胎？ [单选题] *

○是

○否

15. 您是否患有认知功能障碍或心理疾病？ [单选题] *

○是

○否

16. 您是否患有恶性肿瘤、急性或亚急性重型肝炎、重症再生障碍性贫血、特发性血小板减少性紫癜、急性阑尾炎、急性胰腺炎、TORCH 综合征等重大疾病？ [单选题] *

○是

○否

17. 您是否患有慢性疾病，如糖尿病控制不佳、冠心病、高血压控制不佳等 [单选题] *

○是

○否

18. 您最近一周是否患有急性胃肠道疾病？ [单选题] *

○是

○否

19. 您是否在孕前已出现恶心呕吐症状并持续到现在？ [单选题] *

○是

○否

20. 您是否患有其他引起恶心呕吐症状的疾病？如葡萄胎 [单选题] *

○是

○否

21. 您近一周是否在服用维生素 B6、抗敏安、胃复安、昂丹司琼、泼尼松等药物？ [单选题] *

○是

○否

您的姓名： [填空题] *

____

您的手机号（若问卷显示您初步符合入组标准，我们会与您电话联系进行最后的筛选） [填空题] *

____
